# Alanine Expansions Associated with Congenital Central Hypoventilation Syndrome Impair PHOX2B Homeodomain-mediated Dimerization and Nuclear Import*

**DOI:** 10.1074/jbc.M115.679027

**Published:** 2016-04-27

**Authors:** Simona Di Lascio, Debora Belperio, Roberta Benfante, Diego Fornasari

**Affiliations:** From the ‡ Department of Medical Biotechnology and Translational Medicine, Università degli Studi di Milano, 20129 Milan, Italy and; the § National Research Council (CNR) Neuroscience Institute, 20129 Milan, Italy

**Keywords:** dimerization, homeobox, protein import, protein misfolding, transcription factor, trinucleotide repeat disease, congenital central hypoventilation syndrome, PHOX2A, PHOX2B, mammalian two-hybrid system

## Abstract

Heterozygous mutations of the human *PHOX2B* gene, a key regulator of autonomic nervous system development, lead to congenital central hypoventilation syndrome (CCHS), a neurodevelopmental disorder characterized by a failure in the autonomic control of breathing. Polyalanine expansions in the 20-residues region of the C terminus of PHOX2B are the major mutations responsible for CCHS. Elongation of the alanine stretch in PHOX2B leads to a protein with altered DNA binding, transcriptional activity, and nuclear localization and the possible formation of cytoplasmic aggregates; furthermore, the findings of various studies support the idea that CCHS is not due to a pure loss of function mechanism but also involves a dominant negative effect and/or toxic gain of function for PHOX2B mutations. Because PHOX2B forms homodimers and heterodimers with its paralogue PHOX2A *in vitro*, we tested the hypothesis that the dominant negative effects of the mutated proteins are due to non-functional interactions with the wild-type protein or PHOX2A using a co-immunoprecipitation assay and the mammalian two-hybrid system. Our findings show that PHOX2B forms homodimers and heterodimerizes weakly with mutated proteins, exclude the direct involvement of the polyalanine tract in dimer formation, and indicate that mutated proteins retain partial ability to form heterodimers with PHOX2A. Moreover, in this study, we investigated the effects of the longest polyalanine expansions on the homeodomain-mediated nuclear import, and our data clearly show that the expanded C terminus interferes with this process. These results provide novel insights into the effects of the alanine tract expansion on PHOX2B folding and activity.

## Introduction

Heterozygous mutations in the *PHOX2B* gene lead to congenital central hypoventilation syndrome (CCHS^3^; OMIM ID: 209880), which is characterized by a failure in the autonomic control of breathing and an abnormal ventilatory response to hypoxia and hypercapnia (1).

CCHS patients have a greater predisposition to Hirschsprung disease and neuroblastoma (2, 3) as well as the symptoms of general autonomic nervous system dysfunction (4).

The transcription factor PHOX2B (paired-like homeobox 2b, also known as PMX2B and NBPhox) is a master regulator of autonomic nervous system development (5), and its human orthologue is a 314-amino acid protein that harbors a homeodomain and two polyalanine stretches of 9 and 20 residues, respectively, within the C-terminal domain (6, 7).

The large majority of CCHS patients carry mutations that cause an expansion of the longer polyalanine repeat (polyalanine repeat expansion mutations) (2, 8) ranging from +5 to +13 alanine residues, and it has been reported that there is a correlation between the length of the polyalanine tract and the severity of the respiratory phenotype and autonomic dysfunction (8, 9). Non-polyalanine repeat mutations (*i.e.* missense, nonsense, and frameshift mutations) are less frequent, but they correlate with more severe respiratory symptoms, Hirschsprung disease, and neuroblastoma.

From a functional point of view, it is well established that the homeodomain of PHOX2B is a highly conserved 60-residue region that contains the DNA-binding motif; furthermore, in line with what has been observed in other homeodomain proteins, the PHOX2B homeodomain may also contain nuclear localization signals, be responsible for the formation of homo- and heterodimers (with other homeoproteins, including its paralogue PHOX2A), and establish protein-protein interactions (10). On the contrary, the exact molecular functions of the polyalanine tracts remain largely unknown.

Polyalanine and, more generally, homopolymeric tracts (single amino acid repeats) are common features of eukaryotic proteins and are especially abundant in transcription factors (11, 12). Increasing experimental data show that they can modulate transcription factor activity by acting as flexible spacer elements located between functional protein domains and therefore play a role in protein conformation, protein-protein interactions, and/or DNA binding (13–15). The coding triplet repeat instability that leads to the expansion of these stretches causes a number of human diseases (16, 17), all of which are characterized by protein misfolding that leads to intracellular aggregation, which may be an intrinsic tendency because, beyond a certain threshold, the polyalanine tracts spontaneously form β-sheets *in vitro* (18).

Increasingly long polyalanine tracts also lead to an increased tendency for protein aggregation and possible toxic effects in the case of PHOX2B (19, 20). Nuclear import defects and cytoplasmic aggregation are detectable only in the case of proteins with longer expansions, whereas other defects, such as decreased DNA binding and transcriptional activity, also characterize shorter expansions (19–21). In addition to loss-of-function defects, it has been reported that the mutant protein with the longest expansion (+13 alanines) has a dominant negative effect on the DNA binding and subcellular localization of the wild-type protein (19, 21, 22). Furthermore, the negative effects of PHOX2B mutant proteins on the transcriptional activity of the wild-type protein are promoter-specific (20, 21), but it is not clear if the observed functional effects are the result of direct aberrant interactions between wild-type and mutant proteins and/or with other proteins. It should be noted that the absence of co-aggregation of the wild-type protein with mutants with the shorter expansions, revealed by immunofluorescence, does not exclude the possibility of interactions between the non-aggregated proteins at the molecular level. Because wild-type PHOX2B forms homodimers *in vitro* as well as an important fraction of the mutants with shorter expansions (7, 20), and our previous *in vitro* results suggest the possible formation of non-functional heterodimers (21), we decided to test this hypothesis using a co-immunoprecipitation assay and mammalian two-hybrid system. Furthermore, using the same approaches, we assess the ability of mutated proteins to form heterodimers with PHOX2A. Moreover, given the central role of the homeodomain in DNA binding, nuclear import, and dimerization, we also exploit the effects of the alanine tract expansion on PHOX2B nuclear import process.

## Materials and Methods

### 

#### 

##### Plasmid Construction

All of the oligonucleotides used to generate the constructs are listed in Table 1. The PCR amplifications were performed using the GC-rich PCR system (Roche Applied Science), and all of the DNA fragments obtained by PCR were sequenced on both strands. All of the enzymes used for cloning were purchased from New England Biolabs.

**TABLE 1:** **Nucleotide sequences of the synthetic primers used in the PCR amplifications to generate the deleted and mutant variants of PHOX2B** The enzyme restriction sites used for cloning are underlined.

Construct name	Forward primer (5′–3′)	Reverse primer (5′-3′)
PHOX2B Nter	CGGAATTCCAATGTATAAAATGGAATATTC	GCGAATTCTCACTTGCGCTTCTCGTTG
PHOX2B Nter + HD	CGGAATTCCAATGTATAAAATGGAATATTC	GCGAATTCTCACTCCTGCTTGCGAAAC
PHOX2B HD	CGGAATTCAGCAGCGGCGCATCC	GCGAATTCTCACTCCTGCTTGCGAAAC
PHOX2B HD + Cter	CGGAATTCAGCAGCGGCGCATCC	ATGAATTCGGCTTCCGCCGCAGG
PHOX2B Cter	CGGAATTCTTCGCAAGCAGGAGCGC	ATGAATTCGGCTTCCGCCGCAGG
PHOX2B ΔNLS1	CTCAACGAGACCACTTTCACCAGTGCCCAG	AAAGTGGTCTCGTTGAGGCCGCCGTG
PHOX2B ΔNLS2	GTTCCAGCAGGAGCGCGCAGCG	TCCTGCTGGAACCACACCTGGACTCGC
PHOX2B Δ106–147	ACCACTTTCAACCGCCGCGCCAAG	CGGTTGAAAGTGGTGCGGATGCG
External primers (for ΔNLS1, ΔNLS2, Δ NLS1-2 and Δ106–147)	CACAAGCTTGCTGCGGAATTGTACC	CTCCATTCGCCCCGCAGCTG
GAL4 BD-/ VP16 AD-PHOX2B WT	GGGGTACCATGTATAAAATGGAATATTC	CCGGTACCGGCTTCCGCCGCAGG
GAL4 BD-/ VP16 AD-Nter	GGGGTACCATGTATAAAATGGAATATTC	CCGGTACCTCACTTGCGCTTCTCGTTG
GAL4 BD-/ VP16 AD-Nter + HD	GGGGTACCATGTATAAAATGGAATATTC	CCGGTACCTCACTTGCGCTTCTCGTTG
GAL4 BD-/ VP16 AD-HD	GGGGTACCCAGCGGCGCATCC	CCGGTACCTCACTTGCGCTTCTCGTTG
GAL4 BD-/ VP16 AD-HD + Cter	GGGGTACCCAGCGGCGCATCC	CCGGTACCGGCTTCCGCCGCAGG
GAL4 BD-/ VP16 AD-Cter	GGGGTACCCGCAAGCAGGAGCGC	CCGGTACCGGCTTCCGCCGCAGG
GAL4 BD-/ VP16 AD-PHOX2A	GCGGCCGCCGGGCCGATGGACTACTCCTACC	TTGCGGCCGCCTAGAAGAGATTGGTCTTCAGGGC

##### Expression Plasmids

The MYC-tagged PHOX2B wild-type, +7 alanine, and +13 alanine mutant plasmids have been described previously (19, 21, 22).

The generation of the expression plasmid HA-tagged PHOX2B wild type (HA-PHOX2B WT) has been described elsewhere (21). The PHOX2B deletion constructs (Nter, Nter + HD, HD, HD + Cter, and Cter) were obtained by amplifying specific regions of PHOX2B cDNA using HA-PHOX2B WT as the PCR template. The primers used contain the EcoRI restriction site, and after enzymatic digestion with EcoRI, the PCR products were inserted into the HA-PHOX2B WT vector after the same enzyme had been used to remove PHOX2B cDNA. The HD + Cter +13Ala and the Cter +13Ala constructs were obtained by digesting the corresponding wild-type plasmids containing the normal alanine tract with the PpuMI restriction enzyme. The resulting 270-bp region, which encompasses the alanine tract, was replaced by the 309-bp region obtained using the same enzyme to digest the MYC-tagged PHOX2B +13Ala plasmid (19, 22).

The HA-PHOX2B ΔNLS1, HA-PHOX2B ΔNLS2, and HA-PHOX2B Δ106–147 constructs were made using overlap extension PCR (23) with the HA-PHOX2B WT plasmid as the template. Two chimeric primers were used for each construct, each of which consisted of an annealing fragment derived from one flanking region of the deletion and an anchor fragment derived from the flanking region on the other side of the deletion. The external primers were used to insert the deletions in all of the PCR experiments. The first PCR was performed using 40 ng of supercoiled plasmid containing PHOX2B cDNA to amplify two partially overlapping DNA fragments carrying the deletion. The two purified fragments were then annealed and used as the template (50 ng/each) for a second PCR using the external primers to obtain a single product. The PCR products were cloned after double enzymatic digestion with HindIII/PpuMI in the HA-PHOX2B WT plasmid, and the 270-bp region, which was removed by means of PpuMI enzymatic digestion, was subsequently inserted into the resulting plasmid after digestion with the same enzyme. The HA-PHOX2B ΔNLS1-2 plasmid was obtained by means of the same strategy using the HA-PHOX2B ΔNLS2 plasmid as the template and the PHOX2B ΔNLS1 forward and reverse primers.

The HA-PHOX2B +13Ala, HA-PHOX2B ΔNLS1 +13Ala, HA-PHOX2B ΔNLS2 +13Ala, and HA-PHOX2B ΔNLS1-2 +13Ala constructs were generated by means of enzymatic digestion with PpuMI and the replacement of the region encompassing the two PpuMI restriction sites containing the normal alanine stretch with the expanded stretch.

The MYC- and HA-tagged PHOX2A expression vectors were obtained by cloning human PHOX2A cDNA (24) into the EcoRI site of pCMV-MYC or pCMV-HA (Clontech).

##### The Mammalian Two-hybrid System Plasmids

The CheckMate mammalian two-hybrid system kit (Promega) was used to provide the pBIND vector containing the yeast GAL4 DNA binding domain (BD) and *Renilla reniformis* luciferase under the control of the SV40 promoter, the pACT vector containing the herpes simplex virus VP16 activation domain (AD), and the pG5LUC plasmid containing five GAL4 binding sites upstream of a minimal TATA box and the firefly luciferase gene.

The plasmids encoding GAL4 BD-PHOX2B, VP16 AD-PHOX2B, VP16 AD-Nter, VP16 AD-Nter + HD, VP16 AD-HD, VP16 AD-HD + Cter, and VP16 AD-Cter were generated by means of PCR using primers containing the KpnI restriction site. The HA-PHOX2B WT plasmid was used as the PCR template, and the PCR products were cloned in the pBIND and pACT vectors after KpnI enzymatic digestion.

The GAL4 BD-PHOX2B +7Ala/+13Ala/ΔAla and the VP16 AD-PHOX2B +7Ala/+13Ala/ΔAla mutant plasmids were obtained by means of PpuMI enzymatic digestion of the corresponding wild-type plasmids. The 270-bp fragment between the two PpuMI restriction sites was replaced by the corresponding regions containing the expanded or deleted alanine stretches, which were isolated from the previously described expression plasmids HA-PHOX2B 0Ala, PHOX2B +7Ala (21), and PHOX2B +13Ala (19, 22) by means of enzymatic digestion using the same enzyme.

The plasmids encoding GAL4 BD-PHOX2A and VP16 AD-PHOX2A were generated by means of PCR using primers containing the NotI restriction site. The PHOX2A in pCDNA3 plasmid was used as the PCR template (24), and the PCR products were cloned in the pBIND and pACT vectors after NotI enzymatic digestion.

##### Reporter Plasmids

The *DBH* promoter reporter plasmid construct was obtained by cloning a 993-bp *DBH* regulatory region into pGL4 basic vector (Promega), as described previously (21).

##### Cell Cultures, Transient Transfections, and Luciferase Assays

The HeLa cells were grown in Dulbecco's modified Eagle's medium (Lonza), and the SK-N-BE(2)C cells were maintained in RPMI 1640 without l-glutamine (Lonza). Each medium was supplemented with 10% fetal calf serum (PAA Laboratories), 100 units/ml penicillin (Lonza), 100 μg/ml streptomycin (Lonza), and 2 mm l-glutamine (Lonza).

The cells were transiently transfected or co-transfected by means of lipofection (FUGENE HD, Promega) as described previously (21, 25, 26) using 1.5 × 10^5^ SK-N-BE(2)C or 5 × 10^4^ HeLa cells.

In mammalian two-hybrid experiments, 80 fmol of each expression vector were combined with 160 fmol of pG5LUC plasmid. The luciferase assay was carried out using the Dual-Luciferase reporter assay system (Promega) as described previously (26, 27). All of the transfections were performed in triplicate, and each construct was tested in at least three independent experiments using different batches of plasmid preparation. The numbers of independent transfection experiments are indicated in the figure legends.

##### Immunofluorescence

Immunofluorescence was performed as described previously (25). HeLa cells plated on 1.7 × 1.7-cm^2^ glass coverslips were grown to 50% confluence and transfected with the expression plasmid of interest (160 fmol of DNA). The HA-tagged PHOX2B proteins were detected by means of primary rabbit anti-HA antibody (1:50; Sigma, catalog no. H6908) and the secondary Alexa Fluor 488 anti-rabbit antibody (1:400; Invitrogen, catalog no. A11034).

The GAL4 BD and VP16 AD fusion proteins (except for VP16 AD-Nter, VP16 AD-Nter + HD, and VP16 AD-HD) were analyzed using primary chicken anti-PHOX2B antibody (1:100 (25)) and the secondary DyLight 549-conjugated donkey anti-chicken antibody (1:200; Jackson ImmunoResearch, Inc. (West Grove, PA), catalog no. 703-505-155). The VP16 AD-Nter, VP16 AD-Nter + HD, and VP16 AD-HD proteins were analyzed using mouse VP16 antibody (Santa Cruz Biotechnology, Inc., catalog no. sc-7546) and the secondary DyLight 549-conjugated goat anti-mouse antibody (1:400; Jackson ImmunoResearch, catalog no. 115-505-146). The nuclei were stained with DAPI, and the images were acquired using an LSM 510 Meta confocal microscope (Carl Zeiss, Inc.) with ×63 Nikon Apochromat lenses (1.5 numerical aperture).

**FIGURE 1.**
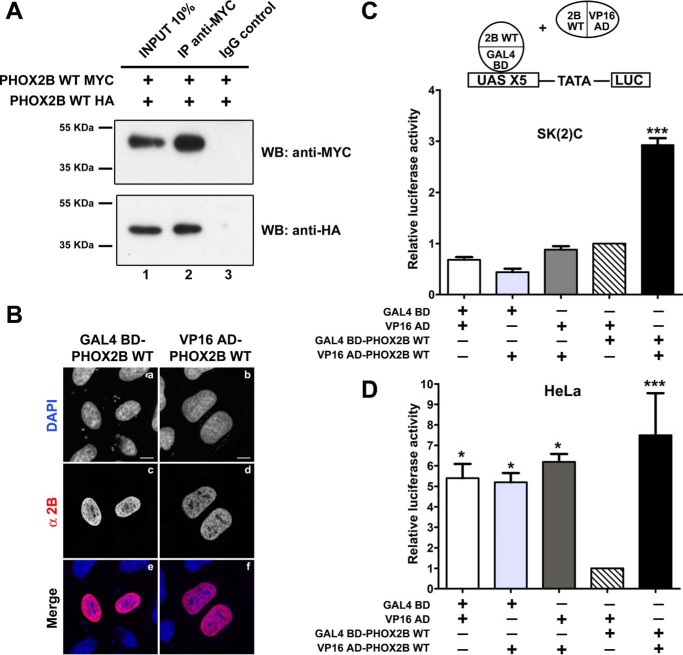
**Homodimerization of PHOX2B wild-type protein.** *A*, representative immunoblotting images of co-immunoprecipitation of MYC- and HA-tagged PHOX2B proteins in transfected HeLa cells. Cell extracts were immunoprecipitated with anti-MYC antibody or control immunoglobulin (IgG) and immunoblotted with anti-MYC (*top*) and anti-HA antibodies (*bottom*). *B*, representative immunofluorescence images of the localization of the GAL4 BD- and VP16 AD-tagged PHOX2B WT fusion proteins in transfected HeLa cells stained using anti-PHOX2B antibody (*c* and *d*). The nuclei were visualized using DAPI (*a* and *b*) and merged with the proteins detected by the anti-PHOX2B antibody (*e* and *f*). *Scale bars*, 10 μm. *C* and *D*, luciferase assays. The *bars* indicate the transcriptional activity of the pG5LUC reporter construct upon co-transfection in neuroblastoma SK-N-BE(2)C (*C*) or HeLa cells (*D*) with a combination of equimolar amounts of a vector containing the cDNA of wild-type protein fused to GAL4 BD (GAL4 BD-PHOX2B WT) and the empty vector containing VP16 AD (*hatched bars*) or with a combination of equimolar amounts of both GAL4 BD and VP16 wild-type fusion proteins (*black bars*). Nonspecific interactions were excluded upon co-transfection of PHOX2B fused to VP16 AD with VP16 AD or GAL4 BD (*light* and *dark gray bars*). The background level of firefly luciferase expression from the pG5LUC vector was determined upon co-transfection with empty vectors containing GAL4 BD and VP16 AD (*white bars*). The results are the means ± S.D. (*error bars*) of the transcriptional activity of the constructs detected in at least three experiments performed in triplicate (*C* and *D*, *n* = 5) and are expressed as -fold increases over the activity of the reporter plasmid co-transfected with the GAL4 BD-PHOX2B WT protein (= 1). *, significant differences from the activity of the wild-type protein fused to GAL4 BD (ANOVA, Tukey's test, *p* < 0.05); ***, significant differences from the activity of the wild-type protein fused to GAL4 BD (ANOVA, Tukey's test, *p* < 0.001).

**FIGURE 2.**
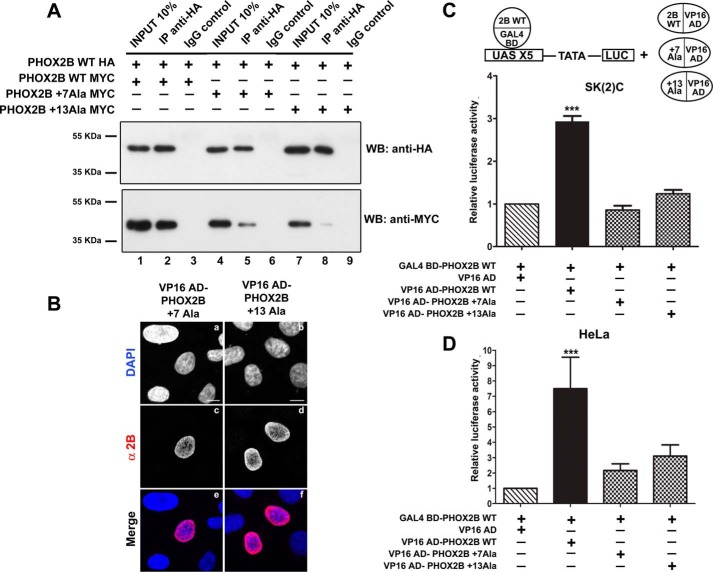
**Heterodimerization of PHOX2B polyalanine-expanded mutants with wild-type protein.** *A*, representative immunoblotting images of co-immunoprecipitation of HA-tagged PHOX2B protein along with MYC-tagged PHOX2B polyalanine-expanded mutants in transfected HeLa cells. Cell extracts were immunoprecipitated with anti-HA antibody or control immunoglobulin (IgG) and immunoblotted with anti-HA (*top*) and anti-MYC antibodies (*bottom*). *B*, representative immunofluorescence images of the localization of the VP16 AD-PHOX2B +7Ala and VP16 AD-PHOX2B +13Ala fusion proteins in transfected HeLa cells stained using anti-PHOX2B antibody (*c* and *d*). The nuclei were visualized using DAPI (*a* and *b*) and merged to the proteins detected by the anti-PHOX2B antibody (*e* and *f*). *Scale bars*, 10 μm. *C* and *D*, luciferase assays. The *bars* indicate the transcriptional activity of the pG5LUC reporter construct upon co-transfection in neuroblastoma SK-N-BE(2)C (*C*) or HeLa cells (*D*) with a vector containing the cDNA of wild-type protein fused to GAL4 BD (GAL4 BD-PHOX2B WT) in combination with the empty vector containing VP16 AD (*hatched bars*), the VP16 wild-type fusion protein (*black bars*), or the VP16-PHOX2B fusion protein carrying polyalanine expansions (*cross-hatched bars*). The results are the means ± S.D. (*error bars*) of the transcriptional activity of the constructs detected in at least three experiments performed in triplicate (*C* and *D*, *n* = 5) and are expressed as -fold increases over the activity of the reporter plasmid co-transfected with the GAL4 BD-PHOX2B WT protein (= 1). ***, significant differences from the activity of the wild-type protein fused to GAL4 BD (ANOVA, Tukey's test, *p* < 0.001).

**FIGURE 3.**
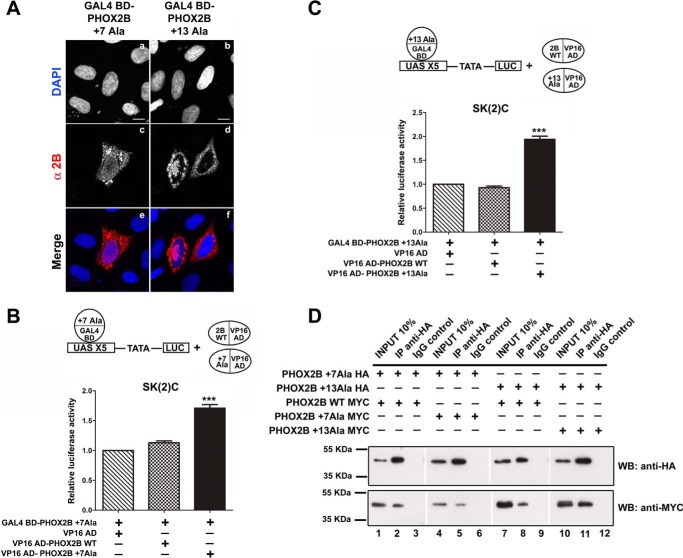
**Homodimerization of PHOX2B polyalanine-expanded mutants.** *A*, representative immunofluorescence images of the localization of the GAL4 BD-PHOX2B +7Ala and GAL4 BD-PHOX2B +13Ala fusion proteins in transfected HeLa cells stained using anti-PHOX2B antibody (*c* and *d*). The nuclei were visualized using DAPI (*a* and *b*) and merged with the proteins detected by the anti-PHOX2B antibody (*e* and *f*). *Scale bars*, 10 μm. *B* and *C*, luciferase assays. The *bars* indicate the transcriptional activity of the pG5LUC reporter construct upon co-transfection in neuroblastoma SK-N-BE(2)C cells with a vector containing the cDNA of PHOX2B +7Ala fused to GAL4 BD (GAL4 BD-PHOX2B +7Ala in *B*) or the cDNA of PHOX2B +13Ala fused to GAL4 BD (GAL4 BD-PHOX2B +13Ala in *C*), in combination with the empty vector containing VP16 AD (*hatched bars*), VP16 wild-type fusion protein (*cross-hatched bars*), or VP16-PHOX2B fusion protein carrying +7 (Fig. 3*B*) or +13 (Fig. 3*C*) alanine expansions (*black bars*). The results are the means ± S.D. (*error bars*) of the transcriptional activity of the constructs detected in at least three experiments performed in triplicate (*B* and *C*, *n* = 4) and are expressed as -fold increases over the activity of the reporter plasmid co-transfected with the GAL4 BD-PHOX2B +7Ala protein or the GAL4 BD-PHOX2B +13Ala protein (= 1). ***, significant differences from the activity of the PHOX2B protein +7 or +13 alanine fused to GAL4 BD (ANOVA, Tukey's test, *p* < 0.001). *D*, representative immunoblotting images of co-immunoprecipitation of HA-tagged PHOX2B polyalanine-expanded mutants along with MYC-tagged PHOX2B wild-type and mutant variants in transfected HeLa cells. Cell extracts were immunoprecipitated with anti-HA antibody or control immunoglobulin (IgG) and immunoblotted with anti-HA (*top*) and anti-MYC antibodies (*bottom*).

All of the immunofluorescence analyses were replicated three times, representing independent transfections, and representative images are shown in Figs. 1, 2, 3, 6, 7, 8, and 9.

##### EMSAs

The EMSAs were performed as described previously (25, 28). The *in vitro* expression of PHOX2A, wild-type PHOX2B, and the deletion and mutant variants was obtained using a commercially available rabbit reticulocyte lysate system (TNT Quick-coupled Transcription/Translation System, Promega), as described previously (25). The oligonucleotides bearing the ATTA 2 and ATTA 3-4 sites of the *PHOX2B* promoter have been described previously (25). The homeodomain binding site oligonucleotide corresponding to the PHOX2B binding site in the *PHOX2A* promoter and the oligonucleotides PRS1 and PBD2 have been described previously (26, 29, 30). All of the oligonucleotides were purchased from Sigma-Aldrich. The antibodies used in EMSA experiments are the rabbit anti-HA antibody (Sigma, catalog no. H6908) and the chicken anti-PHOX2A and PHOX2B antibodies, previously validated and characterized (24, 25). The EMSA experiments were replicated at least two times, using different batches of rabbit reticulocyte lysate.

##### Co-immunoprecipitation Assays and Western Blotting Analyses

HeLa cells (8 × 10^5^) were plated in 100-mm Petri dishes and transiently transfected by means of lipofection (FUGENE HD, Promega) with 700 fmol of each expression vector and harvested after 24 h, when the total extracts were prepared for immunoprecipitation. Proteins were extracted in lysis buffer (25 mm Tris-HCl, pH 8.0, 150 mm NaCl, 1% Nonidet P-40, 0.5% sodium deoxycholate, 1 mm MgCl_2_, 0.2 mm phenylmethylsulfonyl fluoride, Sigma protease inhibitors mixture, and 250 units/ml Pierce universal nuclease for cell lysis (Thermo Fisher Scientific)). Lysates were clarified by 30 min of centrifugation at 16,000 × *g* and 4 °C to remove cell debris and afterward precleared using 20 μl of protein G-agarose bead slurry (Invitrogen) for 1 h under constant rotation. The precleared extracts were incubated overnight at 4 °C with 5 μg of each primary antibody (monoclonal mouse anti-MYC antibody (Sigma-Aldrich, catalog no. M5546), polyclonal rabbit anti-HA antibody (Sigma-Aldrich, catalog no. H6908), and preimmune mouse or rabbit IgG (Santa Cruz Biotechnology, catalog nos. sc-2025 and sc-2027)), and the immunocomplexes were captured by protein G-agarose bead slurry for 4 h at 4 °C with rotation.

**FIGURE 4.**
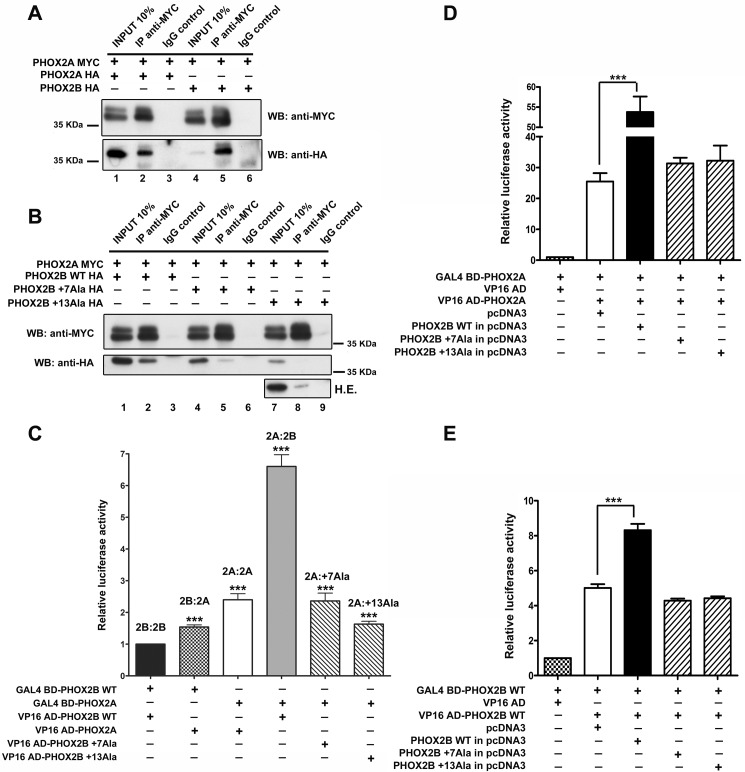
**Homodimerization of PHOX2A protein and its heterodimerization with PHOX2B wild-type protein and with polyalanine-expanded mutants.** *A* and *B*, representative immunoblotting images of co-immunoprecipitation of MYC-tagged PHOX2A along with HA-tagged PHOX2A, PHOX2B (*A*), and PHOX2B polyalanine-expanded mutants (*B*) in transfected HeLa cells. Cell extracts were immunoprecipitated with anti-MYC antibody or control immunoglobulin (IgG) and immunoblotted with anti-MYC (*top*) and anti-HA antibodies (*bottom*). *H.E.*, higher exposure. *C*, luciferase assays. The *bars* indicate the transcriptional activity of the pG5LUC reporter construct upon co-transfection in HeLa cells with a vector containing the cDNA of wild-type protein fused to GAL4 BD (GAL4 BD-PHOX2B WT) in combination with the VP16 wild-type fusion protein (*black bar*) or the VP16-PHOX2A (*cross-hatched bar*); *white*, *gray*, and *hatched bars* indicate the transcriptional activity of the pG5LUC reporter construct upon co-transfection in HeLa cells with a vector containing the cDNA of PHOX2A fused to GAL4 BD (GAL4 BD-PHOX2A) in combination with the VP16-PHOX2A fusion protein (*white bar*), VP16-PHOX2B wild-type fusion protein (*gray bar*), or the VP16-PHOX2B fusion proteins carrying polyalanine expansions (*hatched bars*). The results are the means ± S.D. (*error bars*) of the transcriptional activity of the constructs detected in at least three experiments performed in triplicate (*n* = 4) and are expressed as -fold increases over the activity of the reporter plasmid co-transfected with the GAL4 BD-PHOX2B WT protein in combination with the VP16-PHOX2B wild-type fusion protein (= 1). ***, significant differences from the luciferase activity due to the wild-type protein homodimerization (ANOVA, Tukey's test, *p* < 0.001). *D* and *E*, luciferase assays. The *bars* indicate the transcriptional activity of the pG5LUC reporter construct upon co-transfection in HeLa cells with a vector containing the cDNA of PHOX2A fused to GAL4 BD (GAL4 BD-PHOX2A) in combination with the VP16-PHOX2A fusion protein (*D*, *white bar*) or the cDNA of wild-type protein fused to GAL4 BD (GAL4 BD-PHOX2B WT) in combination with the VP16 wild-type fusion protein (*E*, *white bar*). *Black* and *hatched bars* indicate the transcriptional activity of the pG5LUC reporter upon the co-transfection of the above plasmids with equimolar amounts of a vector encoding PHOX2B WT (*black bars*) or PHOX2B mutants (*hatched bars*). The results are the mean values ± S.D. (*error bars*) of the transcriptional activity of the constructs detected in at least three experiments performed in triplicate (*D* and *E*, *n* = 3) and are expressed as -fold increases over the activity of the reporter plasmid co-transfected with the GAL4 BD-PHOX2A protein (= 1; *D*) or the GAL4 BD-PHOX2B WT protein (= 1; *E*). ***, significant differences from the luciferase activity due to PHOX2B or PHOX2A homodimerization (ANOVA, Tukey's test, *p* < 0.001).

The beads were collected by centrifugation and gently washed and resuspended in sample loading buffer. The immunocomplexes were dissociated from the beads by boiling the samples and then separated by SDS-PAGE and transferred onto nitrocellulose membrane. Western blotting was performed as described previously (24) using the primary anti-MYC (1:1000; Sigma-Aldrich, catalog no. M5546) and anti-HA (1:500; Sigma-Aldrich, catalog no. H6908) antibodies. All of the co-immunoprecipitation experiments were replicated three times, representing independent transfections, and representative immunoblotting images are shown in Figs. 1–4.

## Results

### 

#### 

##### PHOX2B Forms Homodimers

GST pull-down and gel filtration chromatography experiments have previously shown that wild-type PHOX2B protein forms homodimers *in vitro* (7, 20, 31). To verify these observations in a more physiological context that takes into account the possible influences of the cell environmental factors, such as interactors and post-translational modifications, we tested the ability of PHOX2B to dimerize by co-immunoprecipitation.

To this end, we transiently transfected HeLa cells, which do not endogenously express PHOX2B, with MYC- and HA-tagged PHOX2B encoding plasmids, and when the proteins were immunoprecipitated with the anti-MYC antibody, the precipitated complex also contained HA-PHOX2B variant (Fig. 1*A*, *lane 2*), indicating PHOX2B homodimer formation in mammalian cells. As a control, protein G-agarose beads coated with a preimmune antibody did not show HA signal after immunoprecipitation (Fig. 1*A*, *lane 3*). This positive interaction was confirmed by means of the anti-HA antibody immunoprecipitation (Fig. 2*A*, *lane 2*).

Next, we extended our analysis by examining PHOX2B self-interactions using a mammalian two-hybrid system. Thus, we generated PHOX2B fusion proteins respectively with the yeast GAL4 DNA binding domain (GAL4 BD) and herpes simplex virus VP16 activation domain (VP16 AD).

We first tested their expression and subcellular localization by transfecting GAL4 BD-PHOX2B- and VP16 AD-PHOX2B-encoding plasmids into HeLa cells and analyzed the transfected cells by means of confocal microscopy upon immunohistochemistry with an antibody directed against the PHOX2B C terminus; as shown in Fig. 1*B*, both the GAL4 BD-PHOX2B (*a*, *c*, and *e*) and VP16 AD-PHOX2B (*b*, *d*, and *f*) proteins localized diffusely in the nucleus.

The two fusion constructs were then co-transfected, alone or in combination, together with a reporter construct containing five GAL4 binding sites upstream of a minimal TATA box controlling the firefly luciferase reporter gene (pG5LUC) into neuroblastoma SK-N-BE(2)C (Fig. 1*C*) and HeLa cells (Fig. 1*D*).

When VP16 AD-PHOX2B was co-transfected with the GAL4 DNA binding domain (GAL4 BD) or the VP16 activation domain (VP16 AD) in SK-N-BE(2)C and HeLa cells, no significant increase in luciferase activity was measured in comparison with the empty vectors (GAL4 BD and VP16 AD) (Fig. 1, *C* and *D*, *gray bars versus white bars*), excluding nonspecific positive interactions between PHOX2B and VP16 AD or GAL4 BD.

Also, GAL4 BD-PHOX2B did not show nonspecific interactions with the VP16 activation domain (VP16 AD) because there was little if any increase (in SK-N-BE(2)C cells) or a significant decrease (HeLa cells) in luciferase activity in comparison with the empty vectors (GAL4 BD and VP16 AD) (Fig. 1, *C* and *D*, *hatched versus white bars*). This also indicated that PHOX2B does not autonomously activate transcription by the reporter construct. A decrease in luciferase activity has previously been observed in the case of other paired-type homeoproteins, thus suggesting that direct DNA binding by the homeodomain is required for transactivation activity (32–34).

On the contrary, when both fusion constructs (GAL4 BD-PHOX2B and VP16 AD-PHOX2B) were co-transfected in SK-N-BE(2)C (Fig. 1*C*, *black bar*) and HeLa cells (Fig. 1*D*, *black bar*), there was a 3- and 8-fold increase, respectively, in luciferase activity, and the lower luciferase activity measured in SK-N-BE(2)C cells was probably due to the lower transfection efficiency, in comparison with HeLa cells. Our results thus indicate that PHOX2B homodimers can be efficiently detected by using both a co-immunoprecipitation assay and mammalian two-hybrid system.

##### PHOX2B Polyalanine-expanded Proteins Interact Weakly with Wild-type Protein

Mutant proteins can interfere with the activity of the wild-type protein by forming functionally impaired heterodimers and thus have a dominant negative effect. Much evidence has shown that PHOX2B polyalanine-expanded proteins can easily form aggregates, and this has been reasonably interpreted as a consequence of an increased tendency of PHOX2B to self-interact, as has been observed previously in the case of long polyalanine stretches and homopolymeric tracts in general (35). Previous gel filtration experiments have also shown that an important fraction of the mutants with shorter expansions retains the ability to form dimers, whereas there is virtually no formation of species corresponding to the wild-type dimers in the presence of proteins with increasingly long polyalanine tracts (20), and both *in vitro* and *in vivo* experiments suggest the existence of interactions between wild-type and mutant proteins (20–22).

To test the ability of mutant proteins to heterodimerize, we transiently transfected HeLa cells with HA-tagged PHOX2B WT plasmid in combination with MYC-tagged PHOX2B variants carrying the most frequently identified polyalanine expansion in CCHS patients (+7 alanine) or the longest expansion (+13 alanine) and examined their binding potential by co-immunoprecipitation assays.

Our results revealed that mutant proteins immunoprecipitated with PHOX2B wild-type protein and that there was a progressively weaker interaction of the mutant proteins as a function of the length of the expansion (Fig. 2*A*, *lanes 5* and *8 versus lane 2*). Moreover, we observed a reduced amount of mutant proteins in total cell lysates in comparison with wild-type protein (Fig. 2*A*, *lanes 4* and *7 versus lane 1*), due to their decreased solubility after detergent extraction, as confirmed by the higher proportion of mutant proteins in the insoluble fraction (data not shown and previously reported (22)). To exclude the possibility that the lower signals obtained with the mutants in co-immunoprecipitation experiments were due to the decreased protein solubility and to confirm our data, we extended our analysis by using the mammalian two-hybrid system. Co-transfection experiments of VP16 AD-PHOX2B +7Ala or VP16 AD-PHOX2B +13Ala with GAL4 BD-PHOX2B WT showed no functional interactions between the mutants and wild-type protein in SK-N-BE(2)C cells (Fig. 2*C*, *cross-hatched bars versus hatched bar*) and only slight and not statistically significant interactions in HeLa cells, with respect to PHOX2B WT and VP16 AD (Fig. 2*D*, *cross-hatched bars versus hatched bar*).

Immunofluorescence analysis of HeLa cells transiently transfected with VP16 AD-PHOX2B +7Ala and VP16 AD-PHOX2B +13Ala showed that both mutants localized completely in the nucleus (Fig. 2*B*, *a–f*), thus suggesting that the insertion of the nuclear localization signal encoded by the pACT vector is sufficient to force the mutant proteins into the nucleus, particularly the +13 alanine mutants, which usually have a partial cytoplasmic localization (19–21), and excluding the possibility that the reduced capability of the mutant proteins to interact with the wild-type protein may be due to their improper localization.

To evaluate the ability of the mutants to form homodimers, we fused GAL4 BD to PHOX2B carrying +7 or +13 alanine expansions. Unlike the VP16 fusion proteins, the GAL4 BD fusion proteins were not completely detected in the nucleus, thus confirming their tendency to mislocalize in the cytoplasm and indicating that the nuclear localization signal encoded by pBIND vector is weaker and unable to counteract their nuclear import defects (Fig. 3*A*, *a–f*).

In SK-N-BE(2)C cells co-transfection experiments along with pG5LUC plasmid, we found a significant increase in luciferase activity, but the strength of the interactions in mutants forming homodimers was weaker than those observed in wild-type homodimers, probably due to the partial mislocalization of the GAL4 BD fusion proteins in the cytoplasm: 1.7- and 2-fold increases in the case of the +7 alanine and +13 alanine mutant homodimers, respectively (Fig. 3, *B* and *C*, compare *black bars* with *black bar* in Fig. 1*C*). Further, it should be noted that our data concerning interactions among mutants may not allow us to distinguish the formation of dimers and oligomers, but the measured luciferase activities apparently correlate with the increasing propensity of the expanded protein to aggregate as a function of the length of the polyalanine tract. Interactions among mutants were confirmed by co-immunoprecipitation (Fig. 3*D*, *lanes 5* and *11*), and the signal obtained with the +13 alanine mutant, compared with its respective input signal, was stronger than that shown by the +7 alanine mutant, thus suggesting, again, a correlation between the increasing formation of oligomers and the length of the polyalanine tract (Fig. 3*D*, *lane 5 versus lane 4* and *lane 11 versus lane 10*).

Because protein-protein interactions can be direction-dependent, we also assessed the dimerization of the mutants and wild-type protein in the opposite orientation, but once again, we observed no functional interactions with the wild-type protein (Fig. 3, *B* and *C*, *cross-hatched bars*). However, in accordance with our previous co-immunoprecipitation experiments and those performed in HeLa cells using the mammalian two-hybrid system, a small proportion of the wild-type protein was immunoprecipitated with both mutants (Fig. 3*D*, *lanes 2* and *8*). These data indicate that the mutant proteins partially form homodimers and interact very weakly with the wild-type protein and that this was unlikely to be due to the incorrect localization of the mutants. However, the partial discrepancy between biochemical and luciferase data did not exclude the possibility that heterodimers are unable to reconstitute a functional transcription factor and thus stimulate transcription from the reporter gene.

##### PHOX2B Polyalanine-expanded Proteins Retain a Partial Ability to Interact with PHOX2A

GST pull-down and EMSA experiments have previously shown that PHOX2B protein and its paralogue PHOX2A form heterodimers *in vitro* (7, 20, 31). Overexpression of PHOX2A with the PHOX2B +13 alanine mutant did not show co-aggregation or trapping of PHOX2A in the cytoplasm (20). We decided to test the ability of mutant proteins to heterodimerize with PHOX2A by both co-immunoprecipitation and the mammalian two-hybrid system.

When the MYC-tagged version of PHOX2A together with HA-tagged PHOX2A or PHOX2B WT were overexpressed in HeLa cells, immunoprecipitation with the anti-MYC antibody showed interaction with both HA-PHOX2A and HA-PHOX2B (Fig. 4*A*, *lanes 2* and *5*), thus confirming the formation of PHOX2A homodimers and PHOX2A-PHOX2B heterodimers in mammalian cells. When we tested the ability of PHOX2B mutant proteins to interact with PHOX2A, similarly to our observations on PHOX2B-mutant heterodimer formation, the polyalanine-expanded tract reduced the solubility of PHOX2B mutants in comparison with wild-type protein and even more compared with PHOX2A (Fig. 4, *A* (*lanes 1* and *4*) and *B* (*lanes 1*, *4*, and *7*)) and also their binding to PHOX2A (Fig. 4*B*, *lanes 5* and *8 versus lane 2*). To exclude an effect of the protein extraction procedure on the reduced interaction between PHOX2A and mutant PHOX2B proteins, we tested homo- and heterodimer formation by the mammalian two-hybrid system. To this end, we fused GAL4 BD and VP16 AD to PHOX2A and first measured the ability of PHOX2A to form homodimers and heterodimers with PHOX2B. As shown in Fig. 4*C*, the luciferase activity measured when the GAL4 BD-PHOX2A protein was co-transfected with the VP16-counterpart was >2-fold greater than that obtained using PHOX2B fusion constructs (Fig. 4*C*, compare *white bar* with *black bar*), thus suggesting that PHOX2A forms homodimers more efficiently than PHOX2B. When we assessed the heterodimerization of PHOX2A with PHOX2B, we observed that their interaction is stronger than that measured in PHOX2B homodimers (Fig. 4*C*, *cross-hatched* and *gray bars versus black bar*) and direction-dependent (Fig. 4*C*, *cross-hatched versus gray bar*).

Co-transfection experiments of GAL4 BD-PHOX2A along with VP16-PHOX2B mutant fusion proteins (VP16 AD-PHOX2B +7Ala and VP16 AD-PHOX2B +13Ala) showed that the polyalanine expansions severely affect the ability of PHOX2B to form heterodimers with PHOX2A (Fig. 4*C*, *hatched bars versus gray bar*). Nevertheless, the strength of the interactions measured between PHOX2A and the PHOX2B mutants was comparable with or slightly weaker than that of PHOX2A homodimers (Fig. 4*C*, *hatched bars versus white bar*) and even higher than that of PHOX2B homodimers (Fig. 4*C*, *hatched bars versus black bar*). The above experiments indicate that PHOX2B mutants retain a partial ability to heterodimerize with PHOX2A and, therefore, suggest the possible formation of heterodimers (PHOX2A-PHOX2B mutants) with an efficiency comparable with that of PHOX2A homodimers. To investigate whether the relative strong binding of mutants to PHOX2A might compete with normal PHOX2A homodimerization, we performed competition experiments by co-transfecting equimolar amounts of PHOX2B WT or mutants encoding plasmids together with GAL4 BD- and VP16 AD-tagged PHOX2A. Whereas PHOX2B wild-type protein increased luciferase activity >2-fold (Fig. 4*D*, *black bar versus white bar*), both mutants showed a slight but not significant increase of luciferase activity (Fig. 4*D*, *hatched bars versus white bar*), thus suggesting that PHOX2A homodimer formation is not affected by the presence of PHOX2B mutants. A possible interpretation of the increased luciferase activity in the presence of the wild-type protein is that PHOX2B might stabilize PHOX2A homodimer formation and eventually promote transcriptional complex assembly. Remarkably, a similar increase in PHOX2B homodimer activity occurred in the presence of the wild-type protein (Fig. 4*E*, *black bar versus white bar*), whereas a slight reduction was measured in the presence of PHOX2B mutants (Fig. 4*E*, *hatched bars versus white bar*). Our data thus indicate that PHOX2B mutants do not significantly interfere with either PHOX2B or PHOX2A homodimerization.

##### PHOX2B Polyalanine-expanded Proteins Do Not Interfere with PHOX2A-mediated Transactivation of the DBH Promoter and, Conversely, Interact Synergistically

**FIGURE 5.**
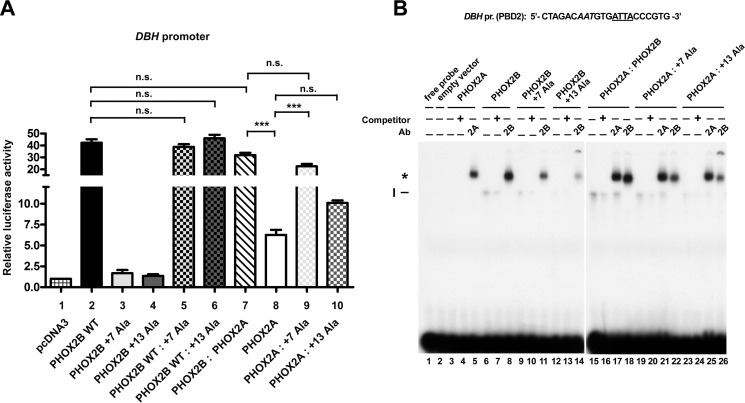
**Molecular effects of co-expressing the polyalanine-expanded mutants and PHOX2A on their transcriptional activity and DNA binding properties.** *A*, luciferase assays. The *bars* indicate the transcriptional activity of *DBH* promoter reporter construct upon co-transfection in HeLa cells with expression vectors containing the cDNA of PHOX2B WT protein (*bar 2*) or of PHOX2A (*bar 8*) or carrying the expanded polyalanine tracts (*bars 3* and *4*); *bars 5*, *6*, *7*, *9*, and *10* indicate the transcriptional activity of *DBH* promoter reporter construct upon co-transfection in HeLa cells with a combination of equimolar amounts of the indicated expression vectors. pcDNA3 indicates the empty vector used as negative control (*bar 1*). The results are mean values ± S.D. (*error bars*) of the transcriptional activity of the constructs of at least three experiments performed in triplicate (*n* = 3) and are expressed as -fold increases over the activity of the reporter plasmid co-transfected with the empty vector (= 1). ***, significant differences from the luciferase activity of the reporter plasmid co-transfected with PHOX2A-encoding vector (ANOVA, Tukey's test); *n.s.*, not significant (ANOVA, Tukey's test). *B*, gel shift assays using the oligonucleotide probe corresponding to a region of the *DBH* promoter containing the ATTA core motif known to bind PHOX2A and PHOX2B. *Top*, sequence of the oligonucleotide used as probe; the ATTA core motif is *underlined*, and the incomplete motif is in *italic type*. The labeled probe was incubated with *in vitro* expressed PHOX2A (*lanes 3–5*), PHOX2B wild-type protein (*lanes 6–8*), PHOX2B +7Ala (*lanes 9–11*), PHOX2B +13Ala proteins (*lanes 12–14*), or a combination of equimolar amounts of the indicated proteins (*lanes 15–26*). The *in vitro* translated pcDNA3 empty vector was used as a control to exclude nonspecific interactions (*lane 2*). The competitions were carried out by adding a molar excess of unlabeled oligonucleotide (*lanes 4*, *7*, *10*, *13*, *16*, *20*, and *24*). Supershift experiments were performed by preincubating the *in vitro* expressed proteins with anti-PHOX2A (*lanes 5*, *17*, *21*, and *25*) or anti-PHOX2B antibodies (*lanes 8*, *11*, *14*, *18*, *22*, and *26*). The *Roman numeral* and the *asterisk* on the *left* indicate the specific retarded complexes obtained and the supershifted complexes containing PHOX2A or PHOX2B. The free probes are shown at the *bottom* of the gels.

Given that PHOX2B mutants retain a partial ability to heterodimerize with PHOX2A, we tested whether PHOX2B mutants might alter PHOX2A-mediated transactivation of the *DBH* (dopamine-β-hydroxylase) promoter, a well characterized PHOX2A and PHOX2B target gene (7, 29, 30). As reported previously (19–21), the mutant proteins showed a marked reduction in their ability to induce the correct activation of the *DBH* reporter construct (Fig. 5*A*, *bars 3* and *4 versus bar 2*), and co-transfection of the polyalanine-expanded proteins with the wild-type protein led to luciferase activity similar to that of the normal PHOX2B alone (Fig. 5*A*, *bars 5* and *6 versus bar 2*). When the *DBH* reporter construct was co-transfected together with PHOX2A, we measured a greater induction by PHOX2B WT protein in comparison with PHOX2A (Fig. 5*A*, *bar 2 versus bar 8*), different from that previously reported (7, 29, 30); this discrepancy could possibly be due to the use of a different regulatory region (993 bp *versus* 232 bp long) and/or reporter plasmid (pGL4 *versus* pGL3 vector). Moreover, the combination of PHOX2A and PHOX2B increased *DBH*-driven luciferase activity to a slightly (but not significant) lower extent than that obtained with PHOX2B alone (Fig. 5*A*, *bar 7 versus bar 2*), confirming that the two factors act independently on the *DBH* promoter (7).

Interestingly, the luciferase activity measured when PHOX2A was co-transfected with the +7 alanine mutant protein was lower but more comparable with that obtained using the wild-type counterpart (23- and 32-fold over the empty vector, respectively), suggesting a (novel) synergistic interaction between the two proteins (Fig. 5*A*, *bar 9 versus bar 7*). A lower synergistic interaction was observed by combining the +13 alanine mutant with PHOX2A (Fig. 5*A*, *bar 10*) although not statistically significant.

Because PHOX2B mutants showed diminished DNA binding (20, 21), we tested by EMSAs whether the observed transcriptional effects correlated with alterations in their DNA binding properties in the presence of PHOX2A; we used the oligonucleotide corresponding to the site in the *DBH* promoter to which PHOX2A/2B bind as dimers (30). As previously reported with other PHOX2B binding sites (20, 21), the expansion of the polyalanine tract progressively reduced DNA binding (Fig. 5*B*, *lanes 9* and *12 versus lane 6*), with the DNA-+13 alanine mutant complex severely affected and hardly detectable.

The retarded band obtained with the *in vitro* expressed PHOX2A protein was undetectable in this set of experiments (Fig. 5*B*, *lane 3*), probably reflecting the relative low affinity of this consensus site and/or the lower binding ability of the *in vitro* expressed protein compared with the PHOX2A-containing nuclear lysates (24, 30, 36, 37); however, a strong ultraretarded band could be obtained in the presence of the anti-PHOX2A antibody (Fig. 5*B*, *lane 5*).

This effect was not due to the interaction of the antibody with other proteins contained in the reticulocyte lysate, because we had previously shown that both PHOX2A and PHOX2B antibodies did not recognize any protein in the lysate (24, 25), but it is in line with previous evidence showing that the antibody may stabilize the interactions between PHOX2A and PHOX2B and their cognate DNA binding sites (21, 24, 25, 36). No specific band was detectable in the presence of reticulocyte lysate programmed with the empty vector pcDNA3 (Fig. 5*B*, *lane 2*), thus excluding nonspecific interactions of the lysate with this probe.

When PHOX2A was combined with PHOX2B, a more intense retarded band than that with the PHOX2B wild-type protein alone was obtained (Fig. 5*B*, *lane 15 versus lane 6*), and the antibody directed against PHOX2A supershifted a more intense band than that observed using the PHOX2A protein alone (Fig. 5*B*, *lane 17 versus lane 5*), suggesting that heterodimers formed of both proteins have a higher DNA binding affinity. Interestingly, a band is still visible in the presence of the antibody against PHOX2A, indicating that only a fraction of the PHOX2B protein forms heterodimers with PHOX2A (Fig. 5*B*, *lane 17*). Similar to the band obtained with the wild-type protein, the retarded bands observed when the +7 and the +13 alanine mutants were mixed with PHOX2A were more intense than those detected with the expanded proteins alone (Fig. 5*B*, *lane 19 versus lane 9* and *lane 23 versus lane 12*). Notably, more intense supershifted bands were observed using the antibody against the PHOX2B mutants, indicating a partial rescue of the DNA binding of the mutants in the presence of PHOX2A (Fig. 5*B*, *lane 22 versus lane 11* and *lane 26 versus lane 14*).

##### The Dimerization Domain Encompasses the Homeodomain and the C-terminal Region of PHOX2B and Does Not Involve the Alanine Stretch

**FIGURE 6.**
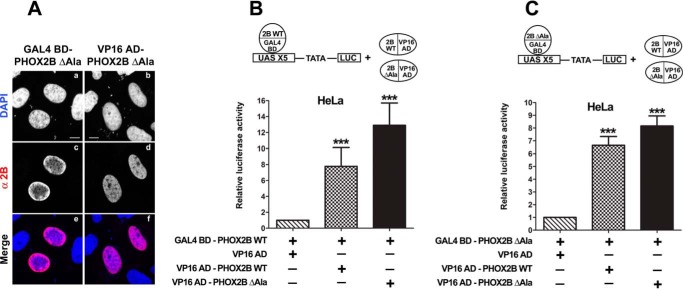
**Homodimerization of PHOX2B protein lacking the polyalanine tract and its heterodimerization with wild-type protein.** *A*, representative immunofluorescence images of the localization of the GAL4 BD- and VP16 AD-PHOX2B ΔAla fusion proteins in transfected HeLa cells stained using anti-PHOX2B antibody (*c* and *d*). The nuclei were visualized using DAPI (*a* and *b*) and merged with the proteins detected by the anti-PHOX2B antibody (*e* and *f*). *Scale bars*, 10 μm. *B* and *C*, luciferase assays of heterodimerization with the WT protein (*B*) or homodimerization (*C*) of PHOX2B ΔAla protein. The *bars* indicate the transcriptional activity of the pG5LUC reporter construct in HeLa cells upon co-transfection with a vector containing the cDNA of wild-type protein fused to GAL4 BD (GAL4 BD-PHOX2B WT; *B*) or a vector containing the cDNA of the deleted protein fused to GAL4 BD (GAL4 BD-PHOX2B ΔAla; *C*) in combination with the empty vector containing VP16 AD (*hatched bars*), VP16 wild-type fusion protein (*cross-hatched bars*), or VP16-PHOX2B fusion protein deleted of the polyalanine stretch (*black bars*). The results are the means ± S.D. (*error bars*) of the transcriptional activity of the constructs detected in at least three experiments performed in triplicate (*n* = 5) and are expressed as -fold increases over the activity of the reporter plasmid co-transfected with the GAL4 BD-PHOX2B WT protein (*B*) or GAL4 BD-PHOX2B ΔAla protein (*C*) (= 1). ***, significant differences from the activity of the wild-type protein fused to GAL4 BD (*B*) or the GAL4 BD-PHOX2B ΔAla (*C*) (ANOVA, Tukey's test, *p* < 0.001).

Despite the predictable central role of the homeodomain in PHOX2B dimerization, because our data from mammalian two-hybrid system experiments indicated that PHOX2B carrying polyalanine-expanded stretches was unable to interact with wild-type protein but at least partially conserved an ability to homodimerize, we estimated the involvement of the alanine tract in dimer formation by generating two constructs expressing a polyalanine-deleted PHOX2B protein fused to the GAL4 BD or VP16 AD domain (GAL4 BD-PHOX2B ΔAla and VP16-PHOX2B ΔAla). As shown in Fig. 6*A*, the deletion of the polyalanine tract did not affect the intracellular localization of the proteins, as previously reported by Di Lascio *et al.* (21). The luciferase activity measured when the GAL4 BD-PHOX2B WT protein was co-transfected with the VP16-polyalanine-deleted protein was comparable with or even slightly greater than that obtained using the wild-type counterpart, thus suggesting that the deleted alanine tract does not affect the ability of PHOX2B to dimerize (Fig. 6*B*, compare *black bar* with *cross-hatched bar*). We likewise also detected strong homodimerization with the GAL4 BD-PHOX2B ΔAla and VP16-PHOX2B ΔAla fusion proteins, thus confirming the small contribution of the alanine stretch to the dimerization properties of PHOX2B (Fig. 6*C*, *black bar*).

**FIGURE 7.**
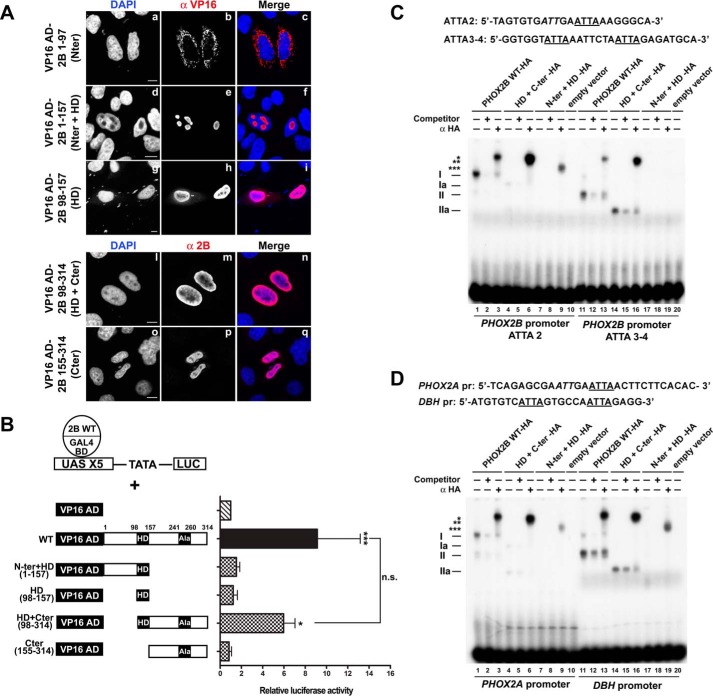
**Mapping of the PHOX2B dimerization domain and contribution of the N- and C-terminal domains to dimerization and DNA binding of PHOX2B.** *A*, representative immunofluorescence images of the localization of the VP16 AD-PHOX2B deleted fusion proteins (VP16 AD-Nter, VP16 AD-Nter + HD, VP16 AD-HD, VP16 AD-HD + Cter, VP16 AD-Cter) in transfected HeLa cells stained using anti-VP16 (*b*, *e*, and *h*) or anti-PHOX2B antibody (*m* and *p*). The nuclei were visualized using DAPI (*a*, *d*, *g*, *l*, and *o*) and merged with the proteins detected by the anti-VP16 and anti-PHOX2B antibodies (*c*, *f*, *i*, *n*, and *q*). *Scale bars*, 10 μm. *B*, luciferase assays. The *bars* indicate the transcriptional activity of the pG5LUC reporter construct in HeLa cells upon co-transfection with a vector containing the cDNA of wild-type protein fused to GAL4 BD (GAL4 BD-PHOX2B WT) in combination with the empty vector containing VP16 AD (*hatched bar*), VP16 wild-type fusion protein (*black bar*), or VP16-PHOX2B deleted fusion protein shown on the *left* (*cross-hatched bars*). The results are the mean values ± S.D. (*error bars*) of the transcriptional activity of the constructs detected in at least three experiments performed in triplicate (*n* = 4) and are expressed as -fold increases over the activity of the reporter plasmid co-transfected with the GAL4 BD-PHOX2B WT protein (= 1; *hatched bar*). *, significant differences from the activity of the wild-type protein fused to GAL4 BD (ANOVA, Tukey's test, *p* < 0.05); ***, significant differences from the activity of the wild-type protein fused to GAL4 BD (ANOVA, Tukey's test, *p* < 0.001); *n.s.*, not significant (ANOVA, Tukey's test). *C* and *D*, gel shift assays using oligonucleotide probes corresponding to two regions of the *PHOX2B* promoter (*C*) or a region of the *PHOX2A* and *DBH* promoters (*D*) containing the ATTA core motifs known to bind PHOX2B. At the *top*, the sequences of the oligonucleotides used as probes; the ATTA core motif is *underlined*, and the incomplete motif is in *italic type*. The labeled probes were incubated with *in vitro* expressed HA-tagged PHOX2B wild-type protein (*lanes 1–3* and *11–13*), PHOX2B HD + Cter (*lanes 4–6* and *14–16*), or PHOX2B Nter + HD proteins (*lanes 7–9* and *17–19*). The *in vitro* translated pcDNA3 empty vector was used as a control to exclude nonspecific interactions (*lanes 10* and *20*). The competitions were carried out by adding a molar excess of unlabeled oligonucleotide (*lanes 2*, *5*, *8*, *12*, *15*, and *18*). Supershift experiments were performed by preincubating the *in vitro* expressed proteins with anti-HA antibody (*lanes 3*, *6*, *9*, *13*, *16*, and *19*). The *Roman numerals* on the *left* indicate the specific retarded complexes obtained using *in vitro* expressed PHOX2B wild-type protein (*I* and *II*) or PHOX2B HD + Cter (*Ia* and *IIa*); the *asterisks* indicate the supershifted complexes containing PHOX2B. The free probes are shown at the *bottom* of the gels.

To map the protein dimerization domain, a series of VP16 fusion constructs containing fragments of PHOX2B protein were generated on the basis of its homeodomain boundaries (Fig. 7*B*). We first used immunofluorescence to check the expression and localization of the deleted proteins. The VP16-PHOX2B 1–157 containing the homeodomain and the N-terminal region (Fig. 7*A*, *d–f*; Nter + HD), VP16-PHOX2B 98–157 corresponding to the homeodomain (Fig. 7*A*, *g–i*; HD), VP16-PHOX2B 98–314 including the homeodomain and the C-terminal region (Fig. 7*A*, *l–n*; HD + Cter), and the VP16-PHOX2B 155–314 construct containing the C-terminal region (Fig. 7*A*, *o–q*; Cter) all predominantly localized in the nucleus. Unexpectedly, the proteins containing the N-terminal region (*i.e.* Nter and Nter + HD) showed a very strong tendency to aggregate in the cytoplasm (Fig. 7*A*, *a–c*) and nucleus (Fig. 7*A*, *d–f*), respectively.

We therefore co-transfected the deleted proteins (excluding the construct containing the N-terminal region because it is localized in the cytoplasm) with the GAL4 BD-PHOX2B WT protein. A significant increase in luciferase activity was observed only with the fragment corresponding to the C-terminal region and homeodomain (2B 98–314), but it was less than that obtained using the full-length protein (Fig. 7*B*, compare *cross-hatched bars* with *black bar*); luciferase activity was comparable with that of the background in the case of the C-terminal fragment alone (2B 155–314) and only 2-fold above the background in the case of the fragment containing the N-terminal region and homeodomain (2B 1–157).

The findings of previous studies of other homeoproteins suggest that the region at the end of the homeodomain (helix III) might be critical for dimer formation (34, 38). Bearing in mind the spotlike distribution of the protein lacking the C-terminal region (Nter + HD) that could interfere with this process, we generated a deletion construct missing the region at the end of the homeodomain (encompassing amino acids 148–155). The obtained protein did not interact with the wild-type protein but, once again, aggregated in the nucleus (data not shown). Furthermore, the luciferase activity of the VP16 AD-HD construct (corresponding to the homeodomain) was comparable with that of the background and that obtained using the C-terminal fragment alone (Fig. 7*B*, 2B 98–157), thus suggesting that the presence and integrity of both domains are required for PHOX2B dimerization. We also found slightly lower luciferase activity in the absence of the N-terminal domain, although not statistically significant compared with that obtained with the full-length protein, thus not excluding the possibility that the N-terminal domain may also play a role in the dimerization process.

We also evaluated the role of the C-terminal and N-terminal domains in dimer formation and DNA binding using EMSAs and radiolabeled oligonucleotides corresponding to the *PHOX2B* promoter region containing ATTA core motifs known to bind PHOX2B (ATTA 2 and ATTA 3-4 motifs) (21, 25), the probe corresponding to the PHOX2B binding site in the *PHOX2A* promoter (26, 39), and the PRS1 oligonucleotide corresponding to a region within domain IV of the *DBH* promoter (29).

Incubation of the wild-type protein with each radiolabeled oligonucleotide caused the appearance of specific retarded bands (complexes I and II) that could be competed by a molar excess of cold oligonucleotide and supershifted by the anti-HA antibody (Fig. 7, *C* and *D*, *lanes 1–3* and *11–13*, *). Conversely, DNA binding of the C terminus-deleted protein (Nter + HD) to all of the probes was severely affected (Fig. 7, *C* and *D*, *lanes 7–9* and *17–19*), although a weak complex could be detected in the presence of the HA antibodies (Fig. 7*C*, *lanes 9* and *19*, ***) with the exception of the ATTA 3-4 motif, which suggests that the antibody may stabilize interactions between the truncated protein and DNA. The behavior of the N terminus-deleted protein (HD + Cter) was different; the migration of complexes I and II was faster because of the smaller size of the protein (complexes Ia and IIa), and the deleted protein retained a partial ability to bind DNA (Fig. 7, *C* and *D*, *lanes 4–6* and *14–16*), with complex Ia being the most affected. The same oligonucleotide shown in Fig. 7*D* (corresponding to a region of the *DBH* promoter and containing two homeodomain binding sites) has previously been used with PHOX2A protein in EMSA experiments (30), and it is worth noting that two DNA-protein complexes were observed: one formed when PHOX2A binds a single site as a monomer and another more retarded complex when two PHOX2A molecules simultaneously bind both sites (probably as monomers). We obtained a similar pattern (complex I and II) using the *PHOX2B* promoter (ATTA 3-4 motifs) and the *DBH* promoter probes, both of which contain two ATTA motifs in tandem position with seven and six intervening bases, respectively (*underlined* in Fig. 7, *C* and *D*), thus suggesting that PHOX2B binds to those sites in the *DBH* and *PHOX2B* promoters mainly as a monomer. Moreover, the authors also showed that PHOX2A binds two other probes as a dimer containing a single motif and a second potential incomplete motif with three intervening bases. Because comparison of the nucleotide sequences of the probes containing the ATTA 2 site of the *PHOX2B* promoter and the homeodomain binding site of the *PHOX2A* promoter revealed the presence of one conserved ATTA motif close to a second incomplete motif (in *italic type* in Fig. 7, *C* and *D*), we can reasonably speculate that the more intense retarded band observed in our experiments was probably formed by the binding of PHOX2B as a dimer (Fig. 7, *C* and *D*, *lane 1*, complex I). This suggests that the HD + Cter protein retained its ability to bind DNA as a monomer (complex IIa) but only partially as a dimer (complex Ia). Interestingly, the antibody against the HA tag fused to the HD + Cter protein supershifted a more intense band than that obtained using the PHOX2B WT-HA protein, thus suggesting that the deletion of the N-terminal domain affected the dimer DNA binding affinity but not its formation (Fig. 7, *C* and *D*, *lane 6*, **). Notably, faint bands were also observed using the Nter + HD protein, thus suggesting that it might form dimers *in vitro* (Fig. 7, *C* and *D*, *lane 9*, ***).

##### The Polyalanine-expanded Tract Interferes with Each Localization Signal and Blocks PHOX2B Homeodomain-mediated Import

Our results show that dimerization is impaired when the polyalanine stretch is elongated. Given that previous experiments have shown that nuclear import and DNA binding are also progressively reduced by polyalanine tract expansion (19–21) and that the homeodomain presumably plays a central role in all PHOX2B functions (*i.e.* nuclear import, DNA binding, and dimerization), we further characterized the role of the C-terminal region in modulating homeodomain activity, focusing our attention on nuclear import process.

**FIGURE 8.**
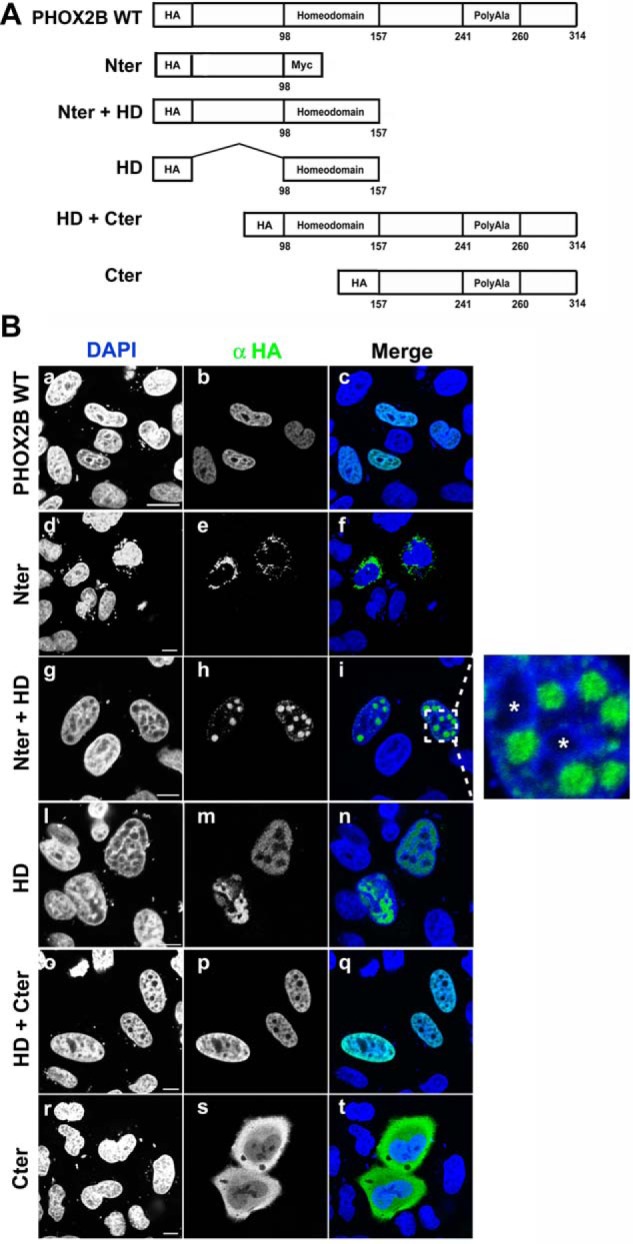
**Contribution of the N- and C-terminal domains to nuclear import of PHOX2B.** *A*, schematic representation of wild-type PHOX2B protein and its truncated constructs. All constructs were fused N-terminally to an HA epitope tag. *Numbers* correspond to the amino acids residues of PHOX2B. *B*, representative immunofluorescence images of the localization of HA-PHOX2B truncated fusion proteins. HeLa cells were transfected with the HA-tagged proteins and analyzed 48 h after transfection by means of immunofluorescence using anti-HA antibody (*b*, *e*, *h*, *m*, *p*, and *s*); the nuclei were visualized using DAPI (*a*, *d*, *g*, *l*, *o*, and *r*) and merged with the proteins detected by the anti-HA antibody (*c*, *f*, *i*, *n*, *q*, and *t*). On the *right* of *i*, an *enlarged view* of the indicated area is shown; the *asterisks* indicate the nucleoli.

**FIGURE 9.**
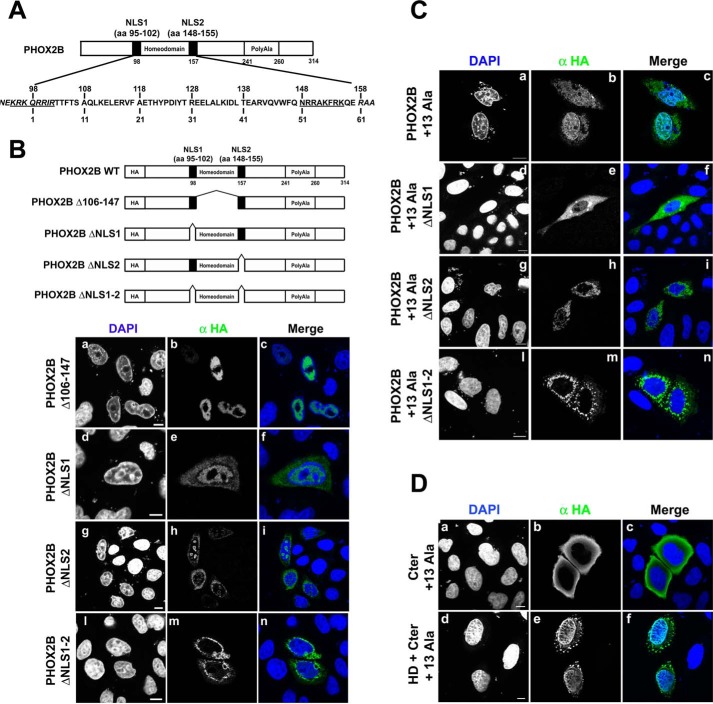
**Identification of PHOX2B NLSs and effects of the polyalanine-expanded tract on PHOX2B nuclear import.** *A*, schematic representation of the PHOX2B protein showing the sequence of the homeodomain and the two putative NLSs (*underlined*). *B*, *top*, schematic representation of PHOX2B carrying deletions of the proximal NLS (ΔNLS1), distal NLS (ΔNLS2), both (ΔNLS1-2), or the entire HD except for the NLSs (Δ106–147). *Black boxes*, NLSs. *Bottom*, representative immunofluorescence images of the subcellular localization of HA-tagged PHOX2B deletion proteins in transfected HeLa cells stained by anti-HA antibody (*b*, *e*, *h*, and *m*); the nuclei were visualized using DAPI (*a*, *d*, *g*, and *l*) and merged with the HA-PHOX2B deleted proteins detected by anti-HA antibody (*c*, *f*, *i*, and *n*). *Scale bars*, 10 μm. *C*, representative immunofluorescence images of the subcellular localization of HA-tagged PHOX2B carrying +13 alanine expansions (PHOX2B +13Ala) and the expanded deletion proteins (PHOX2B +13Ala ΔNLS1, PHOX2B +13Ala ΔNLS2, and PHOX2B +13Ala ΔNLS1-2) in transfected HeLa cells stained by anti-HA antibody (*b*, *e*, *h*, and *m*). The nuclei were visualized using DAPI (*a*, *d*, *g*, and *l*) and merged with the HA-PHOX2B deleted proteins detected by anti-HA antibody (*c*, *f*, *i*, and *n*). *Scale bars*, 10 μm. *D*, representative immunofluorescence images of the localization of the expanded HA-PHOX2B truncated fusion proteins. HeLa cells were transfected with the HA-tagged proteins and analyzed 48 h after transfection by means of immunofluorescence using anti HA antibody (*b* and *e*); the nuclei were visualized using DAPI (*a* and *d*) and merged with the proteins detected by the anti-HA antibody (*c* and *f*). *Scale bars*, 10 μm.

Nuclear localization sequences (NLSs) are quite variable but generally consist of basic residues (40), and in the case of homeodomain proteins, an NLS is often found within or adjacent to the homeodomain itself (41–45). The PHOX2B protein contains two stretches of highly basic amino acids at both ends of the homeodomain (encompassing amino acids 95–102 and 148–155, respectively; Fig. 9*A*). To clarify further the nuclear import of PHOX2B, we identified and characterized PHOX2B NLSs. To confirm the role of the homeodomain in nuclear import of the protein, we cloned the same fragments of the PHOX2B protein (downstream of the smaller HA tag) as those used in the mammalian two-hybrid experiments (Fig. 8*A*) to exclude the possible effects of the heterologous nuclear signal of the VP16 AD tag on protein import and analyzed their subcellular localization by means of immunohistochemistry. We detected cells transfected with all of the truncated constructs except for Nter (corresponding to the N-terminal region), which was only detectable when a MYC tag was cloned downstream of the Nter region (Fig. 8*B*, *panels d-f*), probably because it makes the protein more stable. This confirms that, as previously reported by Wu *et al.* (46), the N-terminal domain of PHOX2B is extremely unstable.

Nuclear staining was only observed when the expressed truncated proteins were those containing the homeodomain and lacking the C-terminal (Nter + HD) or N-terminal region (HD + Cter) (Fig. 8*B*, *g–i* and *o–q*), thus confirming that the HD is required for nuclear localization (47). Moreover, the HD *per se* localized in the nucleus, whereas the Nter and Cter regions were excluded from it, thus indicating that the HD is both required and sufficient for nuclear import (Fig. 8*B*, compare *l–n* (HD) with *d–f* (Nter) and *r–t* (Cter)). It is worth noting that all of the cells transfected with one of the two constructs containing the N-terminal domain (Nter and Nter + HD, Fig. 8*B*, *d–i*) showed very strong cytoplasmic and nuclear aggregation, respectively, thus suggesting that the C-terminal portion of PHOX2B plays a role in keeping the protein in a soluble state, as in the case of the wild-type protein (Fig. 8*B*, *a–c*) and the truncated constructs containing the C terminus (Fig. 8*B*, *o–t*). Remarkably, the peculiar dotlike pattern of the Nter + HD protein showed that the truncated protein is localized in specific foci, corresponding to nuclear regions with weak DAPI staining, which seem to be distinct from the nucleoli surrounded by a characteristic ring of DAPI-positive chromatin (marked with *asterisks* in Fig. 8*B*, *enlarged view* of the indicated area in *i*).

To characterize the role of the two stretches of basic residues at both ends of the homeodomain, we first generated a construct encoding a protein carrying the deletion of the entire HD except for the two putative NLSs (PHOX2B Δ106–147, Fig. 9*B*). As shown in Fig. 9*B* (*a–c*), the deletion did not affect nuclear localization, thus suggesting that the two regions (or at least one) are required for PHOX2B nuclear import. To examine the functional role of each NLS motif in PHOX2B nuclear localization, we introduced deletions into the PHOX2B expression vector that eliminated one or both stretches.

Deletion of the proximal NLS (PHOX2B ΔNLS1) led to nuclear staining of the fusion protein with substantial cytoplasmic fluorescence (Fig. 9*B*, *d–f*), whereas deletion of the NLS2 region (PHOX2B ΔNLS2) led to a more marked cytoplasmic localization (Fig. 9*B*, *g–i*). However, in both cases, the DAPI staining was distorted, and we observed nuclear inclusions corresponding to DAPI-negative regions that resembled the findings obtained when the Nter + HD deletion construct was overexpressed (compare Fig. 9*B*, *d–i*, with Fig. 8*B*, *g–i*).

PHOX2B carrying deletions of both NLS motifs (PHOX2B ΔNLS1–2) accumulated in the cytoplasm around the nucleus (Fig. 9*B*, *l–n*), thus confirming that the concerted action of both regions is required for the nuclear localization of the protein.

To assess the effects of the polyalanine expansions on the homeodomain-mediated nuclear import, we inserted the longest polyalanine expansion (+13) into the constructs carrying the single or double NLS deletion. As shown previously (19–21), the cells transfected with the +13 alanine mutant showed cytoplasmic staining and aggregation as well as nuclear staining (Fig. 9*C*, *a–c*). Unlike the wild type, the combined presence of the expanded polyalanine tract and the deletion of just one NLS (PHOX2B +13Ala ΔNLS1 and PHOX2B +13Ala ΔNLS2) is almost sufficient to completely block protein import (Fig. 9, compare *C* (*d–i*) with *B* (*d–i*)).

Moreover, after deletion of the N-terminal domain, the presence of the C-terminal portion carrying the expanded polyalanine tract directed the homeodomain to a subcellular localization that was different from that of its normal counterpart, which was localized exclusively inside the nucleus (Fig. 8*B*, *o–q*); a fraction of the truncated protein was in the cytoplasm and formed aggregates (Fig. 9*D*, *d–f*), thus suggesting that the expanded C terminus actively interferes with the correct folding of the homeodomain, leading to aggregation. Interestingly, the expanded C-terminal domain localized diffusely in the cytoplasm, like the C-terminal carrying the normal polyalanine tract (Fig. 8*B*, *r–t*), thus indicating that the polyalanine tract *per se* does not massively aggregate (Fig. 9*D*, *a–c*). The above experiments indicate that the nuclear import of the protein is regulated by the homeodomain and that the expanded C terminus interferes with this process.

## Discussion

In the first part of this study, we analyzed the dimerization properties of PHOX2B wild-type and mutant proteins and their possible interactions in a cell model using co-immunoprecipitation and a mammalian two-hybrid system. The analyses confirmed the formation of wild-type homodimers, as already shown *in vitro* (7, 20), and the formation of mutant homodimers/oligomers (although the interactions observed by measuring luciferase activity in mammalian two-hybrid experiments were weaker than those observed in the homodimers of wild-type protein, probably due to the partial mislocalization of the GAL4 BD-tagged expanded proteins) and indicated that expanded PHOX2B mutants interact weakly with wild-type protein.

As in the case of wild type-mutant heterodimers, we detected functional weak interactions only in HeLa cells by the mammalian two-hybrid system, and taking into account that this experimental approach forces proteins into the nucleus and converts an interaction into a defined transcriptional readout, we also considered the possibility that the tendency to aggregation shown by the expanded proteins could interfere with the correct folding of the VP16-tagged expanded proteins or that the formation of heterodimers do not reconstitute a functional transcription factor. However, we reasonably excluded both hypotheses because we were able to detect the formation of mutant homodimers and heterodimers with PHOX2A, although we cannot rule out the possibility that oligomers are formed, because two-hybrid systems have been successfully used to study the oligomerization of aggregation-prone proteins (particularly polyglutamine- and polyalanine-expanded proteins), thus suggesting that this assay is not intrinsically impaired by protein aggregation (35, 48). Interestingly, our data indicate that also the mutant with short (+7) alanine expansion, although still able to partially form homodimers *in vitro* (20), dramatically reduces its ability to dimerize with its wild-type counterpart. Moreover, the same defects have been observed with the mutant carrying the longest expansion (+13 alanine), and the slightly higher luciferase activity measured may correlate with its partial ability to sequester the wild-type protein in the nuclear and cytoplasmic aggregates (19, 21, 22). Therefore, our data exclude the idea that polyalanine-expanded mutants can form strong dimers with wild-type protein and that the formation of non-functional heterodimers may play a major role in CCHS pathogenesis.

In this study, we also analyzed the dimerization properties of PHOX2A and its ability to form heterodimers with PHOX2B wild-type and mutant proteins. Co-immunoprecipitation experiments showed similar interactions among PHOX2B mutated proteins and the wild-type counterpart or PHOX2A, but conversely, the mammalian two-hybrid system showed that PHOX2B and PHOX2A appear to have different interaction properties. Although the homeodomains of PHOX2B and PHOX2A are identical, we measured significantly different strengths of the interactions in the respective homodimers, and interestingly, our data indicate that the formation of PHOX2A-PHOX2B heterodimers is direction-dependent, suggesting a role for other domains of the proteins in modulating these interactions. Several lines of evidence *in vivo* showed that the two proteins are not functionally equivalent, and given that their C-terminal domains are very different and the role of the C-terminal domain of PHOX2B in homodimerization, it is reasonable to suppose that the formation of homo- or heterodimers may be responsible for the creation of different interfaces for differential binding of cofactors. Moreover, the partial ability of PHOX2B mutated proteins to form heterodimers with PHOX2A, with a comparable affinity with PHOX2A homodimers (at least for shorter expansions), suggests the possible presence of species of dimers that differ from conventional homo- and heterodimers.

The hypothesis that PHOX2B mutants may inhibit PHOX2A function in a dominant negative manner was first suggested by Trochet *et al.* (20); however, in that study, PHOX2A nuclear localization was not affected by the overexpression of +13 alanine mutant. Moreover, the recent data reporting specific defects in locus coeruleus development in two human cases of CCHS (one of which carried the +7 alanine mutation) (49) and the finding that PHOX2A is required for LC differentiation (50) have favored, once again, the hypothesis that PHOX2B mutant proteins may exert dominant negative effects on PHOX2A function. Our results with the *DBH* promoter suggest that PHOX2B mutants do not interfere with the transcriptional activity of PHOX2A, but, conversely, PHOX2A is able to synergize with PHOX2B mutants (and this effect is clearly evident at least with the +7 alanine mutant). This observation was unexpected and uncovers additional interesting characteristics of PHOX2B mutants that open the possibility that they may not be simply misfolded and non-functional molecules. Our data indicate first that, despite the reduced transcriptional activity, probably due to the diminished DNA binding, they have a (partial) conserved ability to interact with other components of the transcriptional complex and transcriptional activators and, second, that their defects can be partially counteracted by the interaction with molecules (such as PHOX2A) able to tether them to the promoter of target genes. However, because the nature of the interactors could vary according to the promoter, and we cannot exclude the possibility of new “toxic” interactions, the possible protective role of PHOX2A needs to be further elucidated and investigated. Although we are probably underestimating the interactions among mutants, because of the partial mislocalization of the GAL4 BD-tagged expanded proteins, our findings indicate that the mutants are able to form homodimers, and both the homeodomain and the C terminus are required; therefore, we can reasonably hypothesize that the expansion causes a conformational change in the C-terminal domain that partially blocks interactions between mutant and wild type but not those between mutants (or between mutants and PHOX2A) and that a strong structural constraint on the length of the polyalanine tract is necessary to impose the correct spatial distance and orientation between the homeodomain and the C-terminal region.

Our findings on PHOX2B nuclear import also support the idea of cross-talk between the homeodomain and the C terminus insofar as they show that PHOX2B has two strong functional cooperative NLSs in the homeodomain (a weaker NLS1 in the N-terminal arm and a stronger NLS2 in helix III) and that the polyalanine expansion alters their functionality. In line with this, we found that the polyalanine-expanded tract *per se* does not lead to visible intracellular aggregation because the cytoplasmic signal of the C terminus with the expanded polyalanine tract was diffuse and comparable with that of the wild-type counterpart, and the addition of the homeodomain to the expanded C terminus not only strongly shifts the protein into the nucleus, but also causes its partial aggregation in the cytoplasm. Because the homeodomain itself apparently does not aggregate, this suggests that the expanded C terminus actively interferes with its correct folding. Nuclear transport is a highly regulated process, and the proteins to be transported into or out of the nucleus are bound by transport receptors that recognize the NLSs in the cargo protein. One mechanism regulating protein nuclear import is to modulate the binding affinity of the transport receptor for the NLS cargo, which may occur in various ways: (i) the intermolecular masking of the NLS by a second macromolecule; (ii) the intramolecular masking of the NLS as a result of a post-translational modification within the NLS; and (iii) the intramolecular/interdomain masking of the NLS due to the protein taking on an inhibitory conformation. We favor the idea that the normal C terminus adopts an “open” conformation that allows the homeodomain to function correctly and is then masked by the expanded C terminus in such a way as to prevent dimerization, DNA binding, and nuclear localization. A similar mechanism has been proposed for another homeoprotein containing an alanine stretch (extradenticle, the *Drosophila* homologue of PBX1A), and interestingly, the authors suggest that the stretch itself might be important for maintaining the correct conformation for protein nuclear localization (51). Another possible mechanism is the decreased release in the nucleus of the mutated cargo by transport receptors, as already reported for another homeoprotein (ARX) (52).

Unexpectedly, our findings indicate that, although unstable, the N-terminal region of the protein massively aggregates. Furthermore, the addition of the homeodomain to the N-terminal portion of PHOX2B is sufficient to stabilize it (thus forcing the protein into the nucleus) but apparently does not block the aggregation process. In agreement with our data, it has been shown that the K155X mutant (found in a CCHS patient), which lacks the entire C terminus, aggregates in both the nucleus and the cytoplasm. This is probably because of the absence of the last three residues of the homeodomain, which, as has been previously shown, are important for the NLS2 activity (31). Alternatively, as the truncated proteins accumulate in nuclear regions with weak DAPI staining, which seem to be distinct from the nucleoli surrounded by a characteristic ring of DAPI-positive chromatin, we cannot rule out the possibility that, instead of aggregating, the truncated protein is localized in specific foci. The different pattern observed with the HD + Cter protein, which was indistinguishable from that of the full-length protein, suggests that the C-terminal domain plays a major role in protein solubility and, possibly, protein targeting to the proper subnuclear regions. This is probably a result of greater DNA binding affinity, as supported by the findings of our *in vitro* gel shift experiments showing that the deletion of the C-terminal domain greatly decreases protein DNA binding affinity. In line with this idea is the fact that the two other mutants we tested, which completely lacked DNA binding (*i.e.* PHOX2B ΔNLS1 and PHOX2B ΔNLS2), showed defective subnuclear localization. It has been shown that DNA plays a role in regulating the nuclear distribution of other transcription factors, including members of families of proteins bearing a homeodomain. An intriguing possibility is that, as the DNA binding of polyalanine-expanded proteins progressively decreases as a function of the length of the polyalanine tract, the subnuclear localization of mutants might be slightly different, although by immunofluorescence, the nuclear pattern of proteins with shorter polyalanine expansions is virtually identical to that of wild-type protein. Nuclear import defects and cytoplasmic aggregation are detectable only in the case of proteins with longer expansions, and our previous data indicated that the formation of aggregates is dependent on protein abundance (21, 53). Much evidence regarding other polyalanine proteins, suggests that massive overexpression of the protein might trigger the aggregation process and prevent nuclear import *per se*. Moreover, there are no data on *in vivo* aggregation of PHOX2B protein available, and the debate concerning the involvement of aggregates and their toxicity in CCHS is still open. Nevertheless, our data indicate that the expansion of the polyalanine tract diminishes the efficiency of the homeodomain-mediated protein nuclear import, in comparison with wild-type protein, and together with previous data, our findings indicate that the C-terminal domain is an important modulator of DNA binding, homeodomain-mediated dimerization, and solubility of the protein and that the length of the polyalanine tract is critical to drive the folding of the C-terminal domain, which would in turn influence the spatial orientation of the homeodomain and all of its functions.

Moreover, our data exclude the possibility that the formation of non-functional heterodimers between the wild-type protein and mutants with both short and long expansions may play a major role in CCHS pathogenesis. On the other hand, our findings suggest that PHOX2B mutants may form heterodimers with PHOX2A, with biochemical properties possibly different from those of the homodimers.

## Author Contributions

S. D. L. and D. B. designed, performed, and analyzed the experiments and wrote the paper. R. B. designed and analyzed the experiments. D. F. conceived and coordinated the study. R. B. and D. F. revised the paper critically for important intellectual content. All authors reviewed the results and approved the final version of the manuscript.

## Footnotes

3 The abbreviations used are:

CCHS: congenital central hypoventilation syndrome
BD: DNA binding domain
AD: activation domain
NLS: nuclear localization sequence
ANOVA: analysis of variance
HD: homeodomain
Cter: C-terminal
Nter: N-terminal.
